# Sinus Bradycardia and QT Interval Prolongation in West Nile Virus Encephalitis: A Case Report

**DOI:** 10.7759/cureus.3821

**Published:** 2019-01-03

**Authors:** Mustafa Ajam, Ahmad A Abu-Heija, Mohamed Shokr, Firas Ajam, Ghulam Saydain

**Affiliations:** 1 Internal Medicine, Detroit Medical Center - Wayne State University, Detroit, USA; 2 Cardiology, Detroit Medical Center - Wayne State University, Detroit, USA; 3 Internal Medicine, Jersey Shore University Medical Center, Neptune City, USA

**Keywords:** sinus bradycardia, arrhythmia, west nile virus encephalitis

## Abstract

Cardiac arrhythmias were reported in cases of West Nile Virus (WNV) encephalitis; however, the underlying pathophysiology remains incompletely understood. We present a 67-year-old male with altered mental status, later diagnosed with WNV encephalitis. Hospital course was complicated by progressive sinus bradycardia and corrected QT (QTc) prolongation. These findings persisted despite the absence of classical causes and resolved only after improvement of the underlying encephalitis. After excluding classical causes, autonomic dysfunction is one of the proposed mechanisms behind cardiac arrhythmias in WNV encephalitis. Resolution of arrhythmias is expected after the improvement of underlying encephalitis and should be taken into consideration before proceeding for pacemaker placement or other cardiac intervention. Furthermore, this case highlights the importance of continuous cardiac monitoring in WNV encephalitis patients.

## Introduction

Since its discovery in 1999, West Nile virus (WNV) has been associated with a spectrum of neuroinvasive conditions, including encephalitis, meningitis or acute flaccid paralysis and is the leading cause of viral encephalitis in the United States [1-3].

Despite an improved understanding of this potentially fatal infection, a wide spectrum of its clinical manifestations is yet to be fully understood. Cardiac arrhythmias, including new-onset atrial fibrillation and atrioventricular (AV) node block, have been reported in WNV infections, especially in cases of WNV encephalitis [4-5]. The exact underlying mechanism remains unclear; however, myocarditis or autonomic dysfunction is the proposed explanation for these observed arrhythmias [6]. We report sinus bradycardia and a prolonged corrected QT (QTc) interval resulting in asystole in a previously healthy patient diagnosed with WNV encephalitis. Our case highlights the possible underlying pathophysiology behind cardiac arrhythmias in WNV encephalitis and the importance of continuous cardiac monitoring in such patients.

## Case presentation

**Figure 1:**
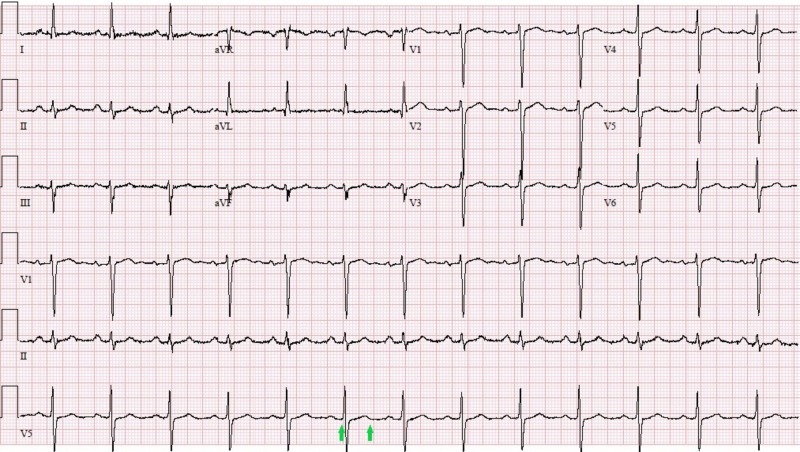
Initial EKG on day one showing normal sinus rhythm and normal QTc interval (426 millisecond); indicated between two green arrows

A 67-year-old male with a history of follicular lymphoma and prostate cancer presented to our hospital with an altered mental status for three days. Physical examination revealed a blood pressure of 146/80 mmHg, a heart rate of 89 beats per minute (BPM), a temperature of 38.4 °C (101.1 °F), normal heart sounds, an inability to follow commands, absent gag reflex and flaccid paralysis involving both upper and lower extremities in addition to absent reflexes in all four limbs. Electrocardiogram (EKG) on day one (Figure 1) showed a normal sinus rhythm and normal intervals.

Few hours following admission, his temperature increased to 39.4 °C (102.9 °F); however, his heart rate remained within the same range at 89 bpm. Initial laboratory investigations revealed acute kidney injury with a creatinine level of 3.2 mg/dL (normal: 0.60-1.20 mg/dL), hypokalemia of 3.2 mEq/L (normal: 3.5-5.1 mEq/L), hypomagnesemia at 1.7 mg/dL (normal: 1.9-2.7 mg/dL), and elevated creatinine phosphokinase level at 4,082 U/L (normal: 35-350 U/L). No significant acid-base disturbances were noted on arterial blood gas (ABG) analysis. Electrolyte imbalances were corrected within the first 48 hours following admission with fluid resuscitation and electrolyte replacement. Initial troponin level was elevated at 0.2 ng/mL (normal: <0.04 ng/mL). White blood cell count was normal at 5,300 cells/µL (normal: 3,500-10,600 cells/µL), with a low lymphocyte count of 600 cells/µL (normal: 1,000-3,800 cells/µL). Head computed tomography (CT) scan did not reveal significant abnormalities. Brain magnetic resonance imaging (MRI) showed small infarcts in the watershed area of deep white cerebral matter. Cerebrospinal fluid (CSF) analysis showed an elevated white cell count of 80 cells/µL with 57% neutrophils and 32% lymphocytes concerning for early viral or bacterial infection. A CSF sample was sent for analysis of WNV immunoglobulin M (IgM) titers, Herpes simplex virus (HSV) polymerase chain reaction (PCR), Epstein Barr virus (EBV) PCR and cytomegalovirus (CMV) PCR. In the meanwhile, intravenous (IV) vancomycin, cefepime, trimethoprim/sulfamethoxazole, and acyclovir were initiated, and the patient was intubated considering his significantly altered mental status and inability to protect his airways.

**Figure 2:**
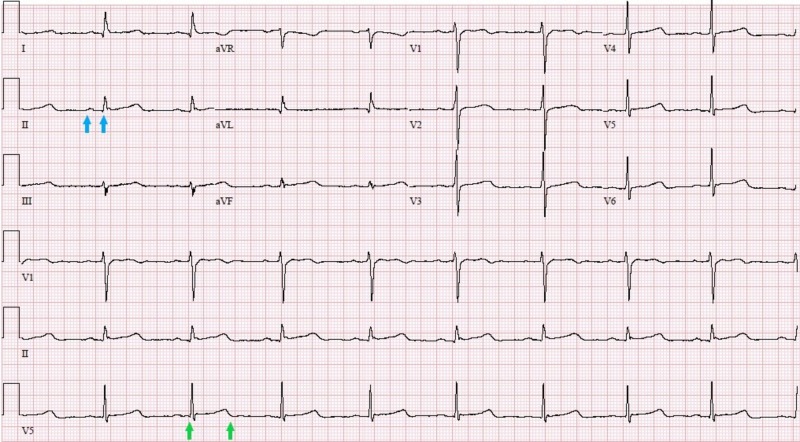
EKG on day five showing first-degree AV block (PR interval of 240 ms, indicated between two blue arrows) and prolonged QTc of 525 ms (between two green arrows) EKG: electrocardiogram, AV: atrioventricular, QTc: corrected QT

On hospital day five, telemetry monitoring revealed a gradual downward trend of the patient’s heart rate, reaching a nadir of 34 bpm. EKG showed sinus bradycardia, first-degree atrioventricular (AV) block and a prolonged QTc interval of 525 ms (Figure 2).

Later that day, the patient suffered from an episode of pulseless electrical activity (PEA), for which cardiopulmonary resuscitation (CPR) was initiated, achieving a return of spontaneous circulation (ROSC) after 11 minutes. Bradycardia and long QTc persisted despite resolution or electrolyte abnormalities, normalization of troponin levels and absence of hypoxia, acid-base disturbances, atrioventricular blocking, or QTc prolonging medications. Furthermore, EKG did not show any regional wall motion abnormalities or evidence of cardiac ischemia.

**Figure 3:**
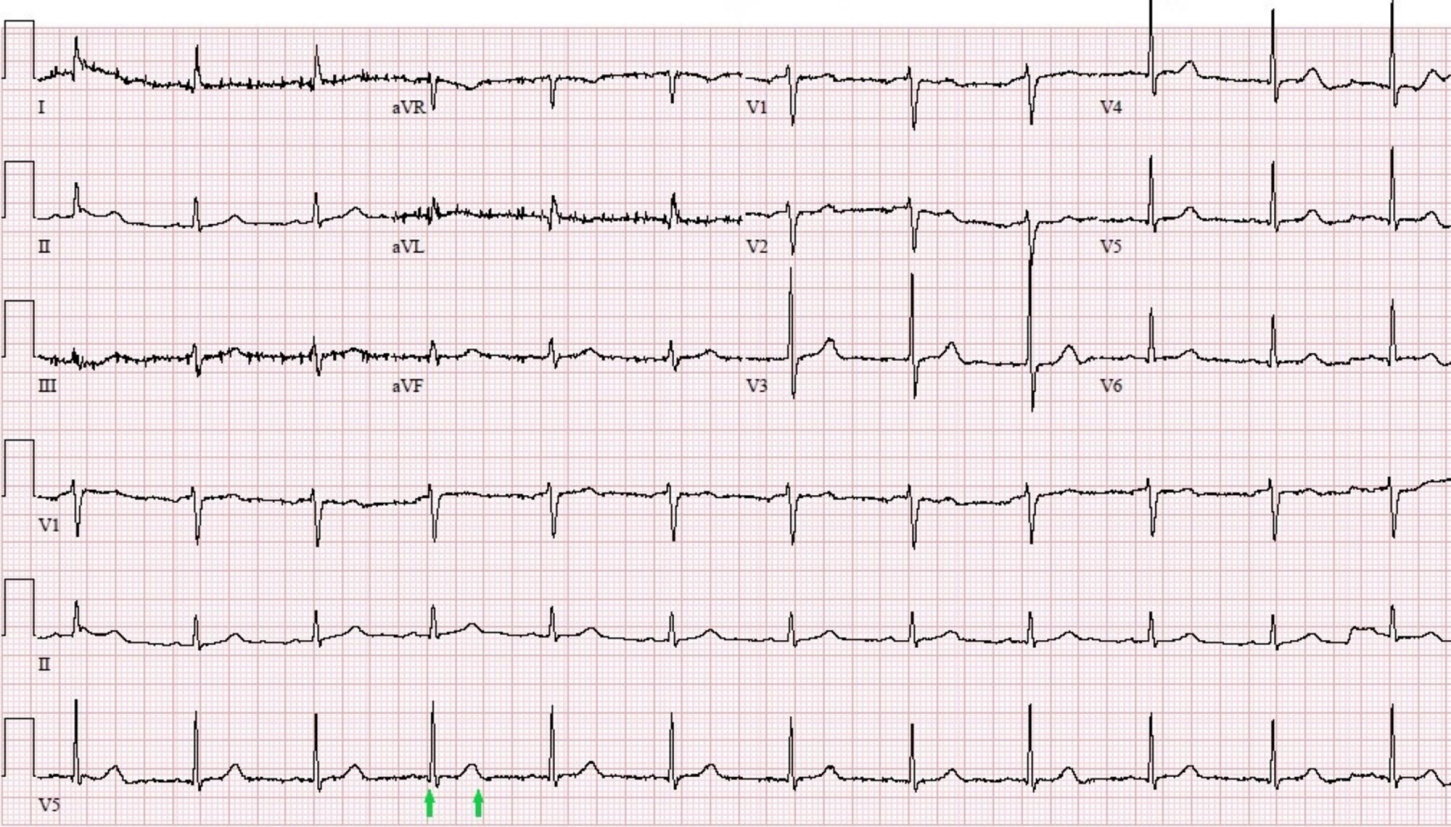
EKG showing resolution of the bradycardia and restoration of normal QTc (439 ms, indicated between two green arrows) EKG: electrocardiogram, QTc: corrected QT

Results for the CSF sample that was sent with an initial lumbar puncture on day one showed positive WNV IgM titers and negative results of HSV, EBV and CMV PCR. IV antimicrobials were stopped and IV immunoglobulin (IVIG) was initiated. In the meantime, cardiology team was consulted for further evaluation and possible pacemaker placement. A decision to proceed with supportive care, IVIG treatment, and continuous monitoring was made before considering pacemaker placement. Three days later, his mental status improved, and the sinus bradycardia and the prolonged QT started to gradually improve (Figure 3) and the need for pacemaker placement was deemed unnecessary at this point.

The patient remained in flaccid paralysis and areflexia and could not be weaned off the ventilator. Tracheostomy and percutaneous gastrostomy tube were placed, and the patient was discharged to a skilled nursing home for rehabilitation.

## Discussion

Less than 1% of cases of WNV infection develop full-blown neuroinvasive disease, presenting with various degrees of neurological involvement, ranging from a mild confusional state to coma and, rarely, even death [2]. In its most severe form, acute flaccid paralysis may develop and can involve diaphragmatic and intercostal muscles leading to respiratory failure [7]. Patients older than 55 years, especially those in an immunocompromised state are at greater risk for neuroinvasive disease [3]. Due to its neurotropism, WNV has a predilection to affect the brainstem structures, especially the medulla and pons, affecting the respiratory and cardiac control centers in that region, leading to various cardiopulmonary complications [2].

Cardiac arrhythmias were described in cases of WNV infections [4-6]. Bode et al. investigated 228 cases of WNV infection in Colorado during 2003 [6]. Cardiac arrhythmias were reported in 7% of the cases. Arrhythmias were more common in the WNV encephalitis group when compared to WNV meningitis and WNV fever. An array of arrhythmias was noted in this study, including new-onset atrial fibrillation as well as worsening of pre-existing atrial fibrillation, second-degree AV block and third-degree blocks [6]. On the other hand, Espinosa et al. reported a case of confirmed WNV encephalomyelitis developing a recurrent idioventricular rhythm requiring permanent pacemaker placement [5]. Kushawaha et al. also reported a patient with WNV encephalitis that developed asystole with resulting death; consequently, autopsy findings revealed evidence of myocarditis secondary to WNV [4]. Table 1 summarizes reported WNV cases associated with cardiac arrhythmia.

**Table 1:** Literature review of reported cases of WNV infection associated with cardiac arrhythmias WNV: West Nile virus, EKG: electrocardiogram, CNS: central nervous system, MRI: magnetic resonance imaging, PEA: pulseless electrical activity

Reference	Case	Initial EKG	Type of CNS infection	Type of rhythm abnormality	Echocardiography findings	Presence of myocarditis	Outcome
Kushawaha et al. [4], 2009	65 years, male	Normal sinus rhythm	Encephalitis	Asystole	Ejection fraction of 50% to 55% along with impaired left ventricular relaxation and dilated left atrium	Autopsy confirmed	Death
Espinosa et al. [5], 2016	65 years, male	Sinus tachycardia	Encephalomyelitis	Idioventricular rhythm resulting in PEA	Hyperdynamic left ventricle with an ejection fraction of 65% and dilated right ventricle	Unknown	Survived after placement of a permanent pacemaker
Our case 2018	67 years, male	Normal sinus rhythm	Encephalomyelitis	Symptomatic sinus bradycardia with prolonged QTc resulting in PEA	Hyperdynamic left ventricle with an ejection fraction of 80%	Unknown. Cardiac MRI could not be performed due to the patient's unstable condition	Survived without cardiac intervention

The exact underlying mechanism behind arrhythmias in WNV virus infection is not well understood. However, the ability of WNV to affect the sympathetic spinal ganglia leading to autonomic nervous system irregularities can play a major role in arrhythmia development [8]. Furthermore, WNV myocarditis is another possible underlying mechanism [4,9]. During the WNV outbreak in Russia in 1999, post-mortem examination of the dead patients revealed WNV myocarditis in 40 cases [9]. However, the total number of deaths was not reported and the percentage of WNV myocarditis among all WNV-related deaths could not be estimated.

In an attempt to expand the literature search criteria, we searched for cardiac arrhythmias in cases of viral encephalitis caused by viruses other than WNV. We found cases of herpes simplex encephalitis associated with cardiac arrhythmias [10-11]. Smith et al. reported a case of sinus node arrest in a patient with herpes simplex encephalitis [10]. Another case of HSV encephalitis presenting with sinus node arrest, attacks of asystole and features of autonomic nervous system abnormalities with labile hypertension, episodic tachycardia, profuse sweating, and hypothermia was also reported by Pollock et al. [11]. Autonomic nervous system abnormalities mediated by the HSV infection were also the proposed mechanism underlying the sinus arrest in both cases [10-11].

Interestingly, our patient also demonstrated another sign of autonomic system involvement, known as pulse-temperature dissociation or relative bradycardia. It refers to an increase in heart rate of less than 10 bpm for every 1 °F increase in core body temperature. Viruses known to be associated with relative bradycardia include, but are not limited to, WNV, Ebola virus, Flaviviridae, and yellow fever virus [12]. Taking into account the resolution of the relative bradycardia contemporaneously with the recovery of the patient’s mental status, we were able to further associate the encephalitis with autonomic dysfunction responsible for the relative bradycardia.

## Conclusions

Although WNV infection primarily affects the central nervous system, cardiac and respiratory complications remain as major contributory factors to mortality in those patients. The mechanism underlying cardiac arrhythmias is not very well understood; however, myocarditis or autonomic abnormalities should be suspected in any case of WNV infection presenting with arrhythmias, especially when other classic causes of arrhythmias are ruled out. Continuous cardiac monitoring is warranted in confirmed or suspected cases of WNV infection, principally in those with a clinical picture of WNV encephalitis. Moreover, the resolution of cardiac arrhythmias in such conditions is expected with the improvement of WNV encephalitis, indicated clinically by improved mental status, which can help avoid unnecessary cardiac interventions such as pacemaker placement.
